# The burden of tetanus in Uganda

**DOI:** 10.1186/s40064-016-2309-z

**Published:** 2016-06-10

**Authors:** Barbara Nanteza, Moses Galukande, Jane Aceng, Joshua Musinguzi, Alex Opio, Anthony K. Mbonye, Eddie Mukooyo, Prosper Behumize, Fredrick Makumbi

**Affiliations:** Ministry of Health, Lourdel Road, P.O. Box 7272, Kampala, Uganda; International Hospital Kampala, Lourdel Road, P.O. Box 8177, Namuwongo, Kampala, Uganda; School of Public Health, College of Health Sciences, Makerere University, Mulago Hill Road, P.O. Box 7072, Kampala, Uganda; School of Medicine, College of Health Sciences, Makerere University, Mulago Hill Road, P.O. Box 7072, Kampala, Uganda

**Keywords:** Tetanus, Safe Male Circumcision, Uganda

## Abstract

**Background:**

The successful scale-up of safe male circumcision (SMC) in Uganda has been hinged on client’s safety and quality of services. However, after the recent three tetanus deaths after circumcision a review of all tetanus cases in one of the hospitals where the cases occurred was initiated. This was to ascertain the potential for an association between tetanus infection and circumcision. Routinely collected national data were also reviewed to determine the burden of tetanus in Uganda and contextualize these incidents.

**Methods:**

A review of medical charts of tetanus cases identified from the inpatients registry at Masafu hospital, Busia district for the period 2009/2010–2013/2014. Data were abstracted from the inpatients registries, charts and HMIS annual reports, and a key informant interview conducted with the in-charge of the ward that treats tetanus patients. All quantitative data were captured in an electronic database. Routine facility data from the National District health Information Software-2 (DHIS-2) for all the 112 districts were also used. Descriptive analysis and Poisson regression models were used for statistical analysis using STATA version 13.0.

**Results:**

Data from the routine DHIS-2 showed a high and increasing burden of tetanus from the emergency/out-patient department records over the 4 year period, highest among females aged 5+ years in all the regions. At the Masafa hospital, the chart review revealed a total of 25 tetanus cases and all were males. Nearly a third (32 %) was aged 7–15 years, with no evidence of circumcision apart from only one case. The rate of tetanus infection among male inpatients over the review period was 2–6 per 1000. The case fatality rate was nearly a half (47.4 %) with deaths occurring within 2 days after admission, and rates of patients’ self-discharge against medical advice were high, 36.8 %. The most common tetanus entry wounds were due to road traffic accidents, followed by diabetic foot. Anti-tetanus serum was only not readily available.

**Conclusion:**

The burden of tetanus is increasing, especially among females aged 5+ years. Tetanus entry wounds among the inpatients in Masafa hospital were mostly due to road traffic accidents, and young males. The tetanus case fatality was very high (47.4 %) and so was patient requested discharge. There is a need to do more to protect the population against tetanus infection, especially among females, and males who need either initial or booster tetanus immunization as SMC is scaled up.

## Introduction

Client safety and quality of circumcision services continue to be the backbone of the scale-up of safe male circumcision (SMC) services in Uganda. Although the uptake of circumcision is still below the 80 % coverage estimated to make the target public health impact of the HIV epidemic, the number has increased from a mere 9325 (0.2 %) in 2010, to slightly over two million (50.1 %) clients as of September 2014 against the 4.2 million circumcisions targets by 2015.

The lower number of adverse events between circumcised clients may provide a reassurance to the potential circumcision clients to seek for SMC services (Kigozi et al. 2008). Use of devices for SMC scale-up including elastic collar compression (PrePex) and collar clamp (ShangRing) have been piloted in Uganda. PrePex device has been assessed for safety in “An Active AE surveillance phase” on 1000 clients and more than 1600 clients in the passive phase as per the WHO guidelines. The AE rate in the PrePex device clients was 1.1 % (0.55, 1.97) during the Surveillance phase and 1.6 % (0.76, 2.90) (Galukande et al. 2014) and 2.6 % (1.18, 4.88) (Kigozi et al. 2014) compared to 2.9 % (2.3, 3.6) observed among clients receiving the surgical method (Kigozi et al. 2008).

Although surgery and PrePex device appear to be safe, four severe adverse events (deaths) were reported in 2014; two deaths from each of these methods (Safety Monitoring Task Force for Safe Male Circumcision report 2014). The cause of death in three adult cases was tetanus (two PrePex clients and one dorsal slit surgical method). A fourth tetanus case in July 2013 after the client had surgical circumcision was not fatal. Therefore the four tetanus cases, three of which were fatal raised concerns about the risk of tetanus in the SMC scale up phase in Uganda.

The SMC death audits for all the three fatal cases provided no direct evidence of the circumcision wound as the portal of entry for the tetanus infections. The confounders included the high background tetanus rates in Uganda, environmental factors, including poor sanitation and personal hygiene, as well as potential occupational hazards (brick laying, metal scrap collection, and riding of ‘motorcycle’ taxis that may expose young men to injuries) and lack of tetanus immunization or boosters may have been key determinants in facilitating the three cases to develop the tetanus infection as shown in some studies (Zziwa 2009). In order to provide safe and quality SMC services, more evidence for the potential association between circumcision and tetanus infection needed to be determined.

Therefore, this study was set out to determine the burden of tetanus infection in Uganda using national HMIS routine facility data, and a review of tetanus medical records from a single health facility. All tetanus cases for the period 2009/2014 from Masafu hospital in Busia were reviewed to provide a better understanding of tetanus patient characteristics.

## Methods

### Study design

This was a cross sectional descriptive study for the medical chart review, and the annual national HMIS records for the 4 years.

### Study setting

Masafu hospital is a government-owned public facility located in Busia district, Eastern Uganda. This hospital works as a referral for all the neighboring districts whose nearest Regional Referral Hospital in Mbale is about 80 km from Masafu. Masafu hospital provides both emergency, outpatient and inpatient services with a total of 95 beds. This facility was chosen for this review because as a district hospital, has been offering surgical SMC services since 2010 and PrePex since August 2014, had a referred circumcised client who died of tetanus infection in September 2014 and would be expected to be a referral site for tetanus cases in the district.

### Data for the burden of tetanus in Uganda

Data were from the DHIS-2 for the years 2011–2014 for all the 112 districts in Uganda. The 112 districts were collapsed into five regions (North, West, East, Central and Kampala). Kampala was grouped separately from Central where it belongs geographically, because it has many facilities that may artificially result in a high number of cases of tetanus for the central region as a whole. A variable reflecting the number of districts per region was constructed for use in the Poisson regression model.

### Data collection

The medical chart review data at the Masafa hospital was conducted by a team of four research assistants. In order to facilitate the quick turnaround for this exercise, the hospital medical superintendent was contacted 5 days prior to the team’s visit to the facility, for permission to work with the records’ officer and other staff who included the SMC focal person from STAR-E [the implementing partner (IP) offering circumcisions in Busia district], the SMC team leader and the Village Health Team member from Masafu hospital. The District Health Officer (DHO) of Busia was also informed about this review with a written communication from the Ministry of Health AIDS Control Program manager, and IRC ethical clearance.

Data sources for this exercise were the hospital SMC registries, patient charts, admission and discharge records and the HMIS monthly and annual reports. All these were recorded on the data abstraction forms previously designed to capture a patient’s socio-demographics, dates of admission and discharge, medical history, presenting complaint, history of presenting complaint, treatment administered and outcome at the time of discharge.

Two independent persons used the inpatient’s registries in the form of black books covering the calendar years 2009–2014 for the identification of tetanus cases. Use of the two independent persons was to ensure that no tetanus case was missed. Information from the registries had patient’s serial number for the calendar year, which was used as part of the patient identification in the abstraction form. Other variables included patient’s sex, age (years), date of admission and discharge, diagnosis with limited clinical notes, and outcome at discharge.

The four independent persons searched tetanus patient’s charts from a pile of over 300 charts, to ensure that no case was left out. The charts were to provide detailed information on patients’ presenting complaint, the nature and cause of wound/injuries (portal of entry for the tetanus infection), treatment administered, and outcome at the time of discharge. The diagnosis of tetanus is clinical and in practice it is hardly confused with any other conditions.

The HMIS annual reports provided data on the number of inpatients disaggregated by sex and age (<5 years or, 5 or older) and by fiscal years 2009/2014. The in-charge of the ward where tetanus patients are admitted was interviewed to elicit information on admission of tetanus patients in the past 12 months, their management while at the facility, and facilities that refer tetanus patients to the hospital, which included a neighboring Dabani Health Facility run by the Catholic Church. All the identified tetanus patients were checked against the PrePex clients’ registry at Masafu hospital using patients’ names, age and village and sub-county of residence to determine if they had received circumcision services.

### Data management

Data for the burden of tetanus were obtained from DHIS-2 for the period 2011–2014. These data were transferred to STATA version 13 of Excel. Data for the medical review were abstracted and captured into an electronic database using EPIINFO developed data entry screens. One person conducted the data entry while another reviewed the electronic file against the abstraction form to independently verify entered data.

### Statistical analysis

#### Burden of tetanus infection using national DHIS-2

Exploratory analysis was conducted and data presented in graphical form showing the number of cases over the 4 years (2011–2014) and by four regions and Kampala (North, West, East, Central and Kampala). Kampala was separated from Central because it has many health facilities that are referrals for the tetanus cases from elsewhere. Further analysis was conducted using Poisson regression models to determine if rates of tetanus cases per region significantly changed over the period 2011–2014. The model adjusted for the number of districts per region, and clustering of observations at the regional level. The 2014 national population census was used as offset for populations at risk in the Poisson regression model to obtain the number of tetanus cases per population.

#### Review of tetanus medical records at Masafu hospital

Descriptive analysis was conducted to generate a mean (SD) and median (inter quartile range) for the continuous data, and proportions for categorical data. Cross tabulations of categorical data with Chi square test and corresponding p values to determine any associations. Differences in continuous variables such as hospital stay in days, and age (years) of tetanus patients by categorical variables were determined by use of non-parametric tests Kruskal–Wallis one-way analysis of variance.

Clinical notes were reviewed and summarized for diagnosis, presenting complaint, history of the presenting complaint, co-morbidities, immunization history, nature and cause of wound/injury (portal of entry) and the location of such injuries, treatment administered and the outcome at discharge. All the quantitative analysis was conducted using STATA version 13.

### Ethical clearance

This review received ethical clearance from the Makerere University School of Public Health Institutional Review Committee (IRC) for exemption of protocol review because all the data are routine and not contacts with the respondents in the field. Permission was also obtained from the District Health Office (DHO) and the hospital Medical superintendent’s office at the hospital.

## Results

### Burden of tetanus infection using national DHIS-2

**Fig. 1:**
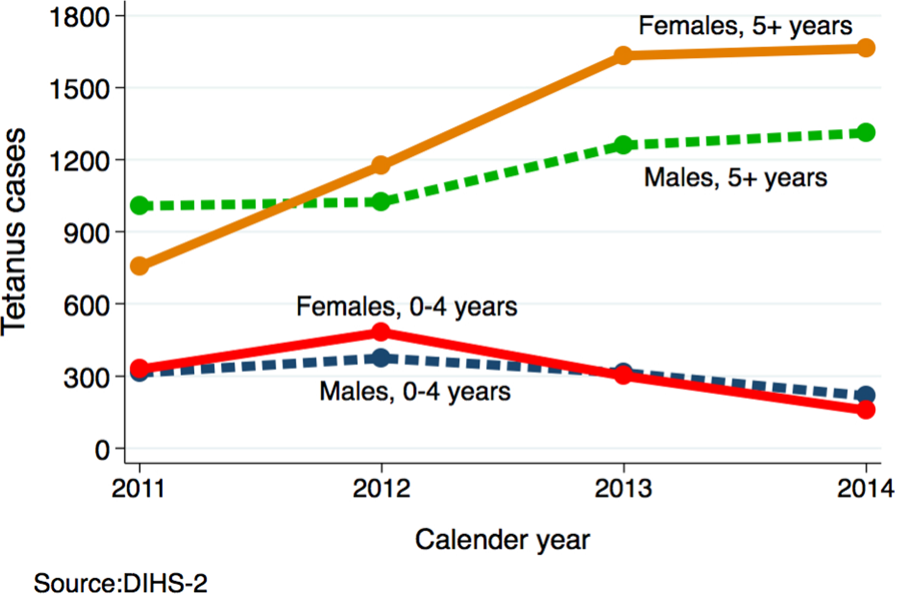
Number of OPD tetanus cases

Table 1 shows the number of tetanus cases by calendar year, sex and age. For the 4 years with data available, the number of recorded tetanus cases through the DHIS2 system has been increasing. Cases aged 5 or more years were three times those aged 0–4 years (which excludes all neonates). Apart from the year 2011, the number of female cases tends to be higher than male cases, and has been steadily increasing overtime for the 5+ years but not 0–4 year olds (see Table 2). Figure 1 shows trends in the changing number of cases over the 4-year period. There is variation in the number of cases by region with Kampala reporting the highest number relative to other regions.

**Table 1:** Number of OPD tetanus cases by year, age and sex country data *Source*: DHIS2

Calendar year	Female		Male	
	0–4	5+ years	0–4	5+ years
2011	329	755	314	1007
2012	482	1176	375	1024
2013	301	1633	314	1260
2014	158	1663	218	1311
Mean (SD)	318 (133)	1307 (430)	305 (65)	1151 (157)

**Table 2:** Number of 5+ years OPD tetanus cases by year, sex and region

Calendar year	Central		Kampala		East		North		West	
	Male	Female	Male	Female	Male	Female	Male	Female	Male	Female
2011	64	136	384	381	66	46	59	184	434	8
2012	60	100	466	474	222	286	120	180	156	136
2013	185	281	549	577	130	139	225	411	171	225
2014	208	229	679	739	110	191	112	254	202	250

**Fig. 2:**
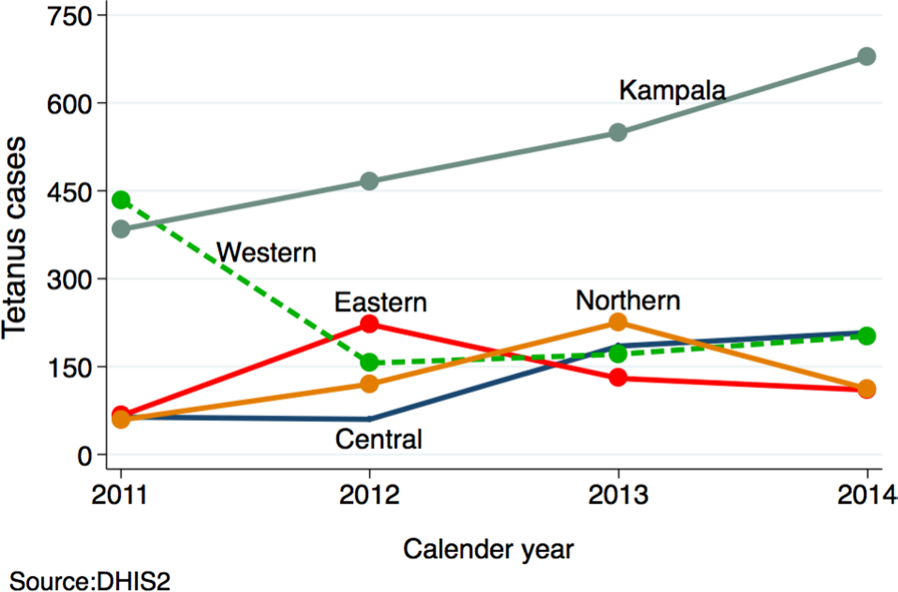
Number of male 5+ years tetanus cases by region

**Fig. 3:**
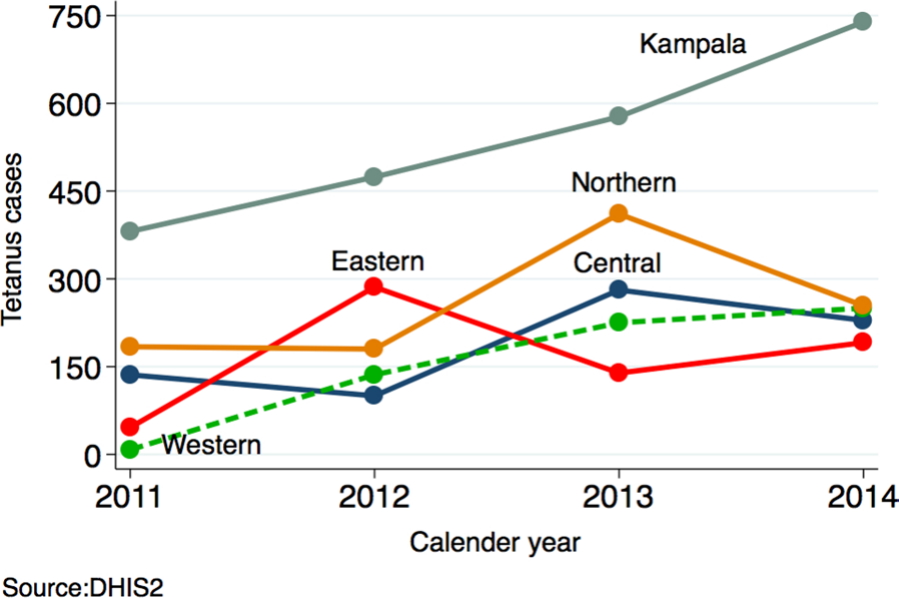
Number of female 5+ years tetanus cases by region

Using the 2014 census population and excluding Kampala, the number of female tetanus cases per population was significantly higher in the years 2013 [adjusted IRR 2.6; 95 % CI (1.51, 4.47)] and 2014 [adjusted IRR 2.18; 95 % CI (0.97, 4.90)] relative to 2011, but no differences were observed among males 5+ years, controlling for clustering at the region level and adjusting for number of districts per region see Figs. 1 and 2. The number of females 5 years and above significantly increased over the years see Fig. 3.

### Social demographics

A total of 25 tetanus patients were identified from the inpatient registries. All patients were males, median (IQR) age 30 (14, 45) years. About a third (32 %) of the patients were 7–15 years, but the majority (40 %) were aged 20–49 years, suggesting that nearly three-quarters (72 %) were in the circumcising age range as per the SMC policy. Almost all patients were from the Samya tribe, a dominant tribe in the region and only 2 were Muslims while others were non-Muslims. Patient’s surnames were used to determine their tribe while the first names provide the best guess of their religion, which was categorized into Muslims and non-Muslims. Five patients were residents of the neighboring districts of Namayingo (4) and Bugiri (1), while the rest were locals of Busia. Just over a third (36 %) of the tetanus patients were identified from FY 2012/2013, and four patients had already been registered in the first half of FY 2014/15.

### Length of hospital stay and outcome at discharge

Only 19/25 patients had information on patients’ duration of hospital stay based on dates on admission and discharge. Hospital stay was longer in the 3 patients discharged healed, median (IQR) 12 (9, 15) days, followed by the six discharged on request 4.5 (2, 8) days, and least for the nine who died 2 (1, 3) days. The patient who escaped from hospital before healing had spent 3 days. The case fatality rate of the tetanus patients at this facility was 47.4 % (9/19) and the self-discharged from the hospital were 36.8 % (7/19). Death rates per 1000 tended to increase over the three fiscal years *FY2010*/*2011* 2.3 (0.28, 8.40), *FY2011*/*2012* 4.2(1.4, 9.72), and *FY2012*/*2013* 6.3 (2.9, 11.9), but declined in the FY2013/2014 2.0 (0.41, 5.76).

### Patient treatment administered

Only five patients had charts with detailed case notes that provided presenting complaint, the nature and cause of wounds/injuries, treatment administered and outcome at discharge. However, only three patients received anti-tetanus serum (20,000 IU). All patients were treated with antibiotics (metronidazole), diclofenac, diazepam, chlorpromazine, Nasal Gastric Tube for feeding and isolation in a dark room. Data on the type and cause of injuries were available in only four cases. The cause of injuries was a road traffic crash (RTC) and wounds located on the lower limbs. All the accidents involved the motorcycle ‘taxi’ (Boda-boda). Two elderly patients aged 59 and 63 years had diabetic foot ulcers.

### PrePex clients

The SMC PrePex register at the facility did not have any of the 25 identified tetanus patients. However, one of the patients who died in September 2014 was a circumcision client from Bugiri hospital, Bugiri district in the neighboring district, about 20 km from Masafu hospital. The audit report conducted in 2014 of the three tetanus deaths following circumcision showed that this patient was safely discharged after PrePex device removal, but later contracted tetanus. His PrePex device placement was August 19th, 2014 and removed a week later on August 26th, 2014 without any health complaint. However, he was admitted to Masafu hospital on September 1st, and died September 2nd, 2014. His profile indicated that he was a bricklayer, a job he continued to do after placement of the PrePex.

### Treatment details of the four tetanus cases with available data

#### Case 1

Patient (A79 0R), aged 14 years, involved in a RTC, reported to hospital on December 26th 2011 and received treatment for injuries. Five days later, on December 31st, 2011 reported back to hospital with stiff neck, and was diagnosed with tetanus. Treatment administered included anti-tetanus serum (20,000 IU), injection PPF, diazepam, diclofenac, but he died on January 2nd 2012.

#### Case 2

Patient (A68 WJ), aged 12 year reported at 10:11 p.m. admitted December 28th, 2011 with injuries due to RTA and with classical signs of tetanus infection. Anti-tetanus serum was not provided because it was unavailable. He was treated with chlorpromazine, and diazepam but died the following day of the December 29th, 2011.

#### Case 3

Patient (A092 WA), aged 14 years was involved in a RTC, admitted on November 29th, 2012 presented with stiff neck and unconscious. Treatment provided included anti-tetanus serum (20,000 IU), inj PPF 0.8 mu once a day for 7 days, diazepam and diclofenac but he died on December 5th 2012.

#### Case 4

Patient (B08 BH), aged 17 years, admitted on August 2nd, 2014 presented with generalized tonic–clonic convulsions, frequent spasms and difficulty swallowing 3 days after he was involved in RTC. Treatment did not include anti-tetanus serum because it was unavailable. He received ceftriaxone, metronidazole, chlopromazine and diazepam but died on August 5th 2014.

The interview with the ward in-charge indicated that tetanus patients sought better treatment from elsewhere, thus the high self-discharge rate. The anti-tetanus serum is usually unavailable due to high cost, and so patients usually request for early discharge if they observe no improvement in their health. For the three patients who escaped from the facility the KII indicated that isolation of patients in the dark room created stigma and the fear of death.

## Discussion

The high and increasing burden of tetanus was observed from the routine data over the 4 years under study. The characterization of tetanus patients in Masafu hospital, a non-traditionally circumcising setting had majority of patients from the local tribe of Samya, only males, nearly a third (32 %) were aged 7–15 years, and with no evidence that circumcision provided the portal of entry apart from only one patient who was referred from Bugiri, a neighboring district. The rate of tetanus infection ranged from 2 to 6 per 1000 among male inpatients for the 4 years reviewed. The case fatality rate was nearly a half (47.4 %) with deaths occurring within 2 days after admission. Self-discharge against medical advice from the hospital was high, 36.8 %. The most common tetanus entry wounds were due to road traffic accident injuries, followed by diabetic foot ulcers reported in two elderly patients with diabetes (a sign of non communicable disease). Treatment with anti-tetanus serum was only available and offered to three of the five patients, no intensive care services were available. These highlight the limitation to appropriate care.

The high and increasing burden of tetanus observed from the routine data over the 4 years under study is of concern. The tetanus infection observed from the SMC implementation may be associated with the already high background rates with tetanus. This is supported by the higher number of tetanus cases among females, who do not receive SMC. Although guidelines exist for tetanus prevention for childhood immunization, prophylaxis against neonatal tetanus, and vaccination against adult tetanus (school going children and high risk groups such as farm workers, military personnel, miners and road traffic accident victims), these may not be followed for reasons including availability of the vaccines in the facilities (National Guidelines on Management of Common Conditions 2012). The high burden reflects poor immunization uptake, which may either be due to poor seeking behaviors or lack of the tetanus toxoid vaccines.

Studies conducted in West Africa showed circumcision (traditional circumcision is very common in West Africa) as being common among tetanus patients (Sow et al. 1993; Soumaré et al. 2005, 2008). However, our data do not provide evidence for this association in this non-traditional circumcising setting. Findings from our review show only one patient contracted tetanus after circumcision procedure. This patient had received SMC in the neighboring district of Bugiri, and at the time of PrePex device removal, he reported no symptoms of tetanus infection. The circumcision status of the two Muslims patients could not be confirmed, but it is unlikely that they were recent converts.

The wounds that may have been the portal of entry of the tetanus infection were due to accidents as a result of motorcycle ‘taxis’ (Boda-boda) in the four cases, and diabetic foot ulcers in two others. Lack of patient records in the majority of patients was a key limitation in having a clearer understanding this important factor. However, given his occupation (as bricklayer) he may have had repeated skin breaches during the course of his work.

A substantial number of tetanus patients were involved in motorcycle taxis, which is consistent with a study conducted elsewhere (Bankole et al. 2012). In Uganda the motorcycle taxis have the highest risk of involvement in RTC (Uganda Bureau of Statistics 2014). Unfortunately, motorcycle taxis are part of the most at risk population for HIV infection and therefore HIV prevention programs, including SMC tend to pay special attention to this group (Linda et al. 2015). Thus, their double burden of the potential for tetanus infection and HIV, requires increased HIV prevention services, especially SMC to pay special attention while screening such clients to inquire about injuries when providing SMC services.

There were no females or children younger than 7 years. The absence of children younger than 7 years may indicate the extent of immunization uptake for children in this setting. Young boys aged 7–15 years reporting with tetanus infection may suggest either non-immunization or waning immunity for tetanus. However, the absence of any females among these cases may suggest less environmental and occupational hazard risk for women and better immunization coverage. Young men in this setting were involved in collecting rusty metal/scrap for trade across the border into Kenya. Such materials may increase their risk of tetanus infection due to the injuries they are likely to sustain in this business (Linda et al. 2015) if immunized with DPT as infants, their immunity may have waned and Uganda has no policy on tetanus booster vaccination. Such occupational exposures and waned immunity may increase their risk of tetanus infection.

The rate of tetanus among males ranged from 2 to 6 per 1000 persons. This is similar to the overall prevalence of tetanus observed in a previous study in a similar setting about 80 Kilometers away from Busia hospital, where the rate was 0.65 % of all admissions. However, Zziwa (2009) study included neonates (Zziwa 2009). This rate may be an underestimate of the actual tetanus prevalence because some cases are not reported.

The high case fatality rate may have been contributed by inadequate services available. Of the five cases with available data on treatment administered, only 3/5 received the anti-tetanus serum considered important in treating tetanus patients. A similar observation was made by Zziwa (2009) where only a third (33.3 %) of cases received anti-tetanus serum mainly due to the unavailability of this medication (Zziwa 2009).

### Study limitations

Data from the routine OPD DHIS2 may contain double reporting because many tetanus cases tend to be referred to higher facilities that are better equipped to manage them, especially the facilities in Kampala and Mulago Hospital. However, there may also be poor health seeking behaviors and some patients never seek medical services, thus never get to be recorded on the system.

The medical records with detailed clinical notes were only available for 5/25 patients while data for the two diabetic foot patients were abstracted from the inpatient registry. The clinical notes also lacked important information such as immunization history, dates of onset of symptoms, and dates when injuries or wounds were sustained, patient’s social demographics (occupation, education, marital status, tribe and circumcision status), and other factors such as personal hygiene and wound care practices.

The role of immunization and circumcision status in tetanus infection could not be determined by the design of medical record review in this setting due to poor data completeness.

Nonetheless, this review provided some insights on how best to implement a more detailed study that addresses the gaps in the data. Better reporting over time may make it appear like the cases are increasing.

## Conclusion

The burden of tetanus is increasing, especially among females aged 5+ years. Kampala recorded highest tetanus cases because it has the national referral hospital and other facilities with specialized care.

In Masafa hospital, tetanus entry wounds were mostly due to road traffic accidents, and among young males. Nearly half of the in-patient tetanus cases did not survive, and self-discharge was common. Access to critical care services such as dedicated ICU was limited or non existent.

Stepping up protection of the population against tetanus infection with either an initial or booster tetanus immunization is urgently needed.

## Abbreviations

DHIS-2: District Health Information Software-2
DHO: District Health Officer
ICU: intensive care unit
IP: implementing partner
MoH: Ministry of Health
OPD: out-patient department
RTA: road traffic accidents
RTC: road traffic crash
SMC: safe male circumcision
